# Supplementary material for: An Aux/IAA Family Member, RhIAA14, Involved in Ethylene-Inhibited Petal Expansion in Rose (Rosa hybrida)
Source: Genes (Basel). 2022 Jun 10;13(6):1041. doi: 10.3390/genes13061041 (PMC9222917; doi:10.3390/genes13061041)
Supplement: Supplementary file 1 [file genes-13-01041-s001.zip › Figures S1-S3.pdf]

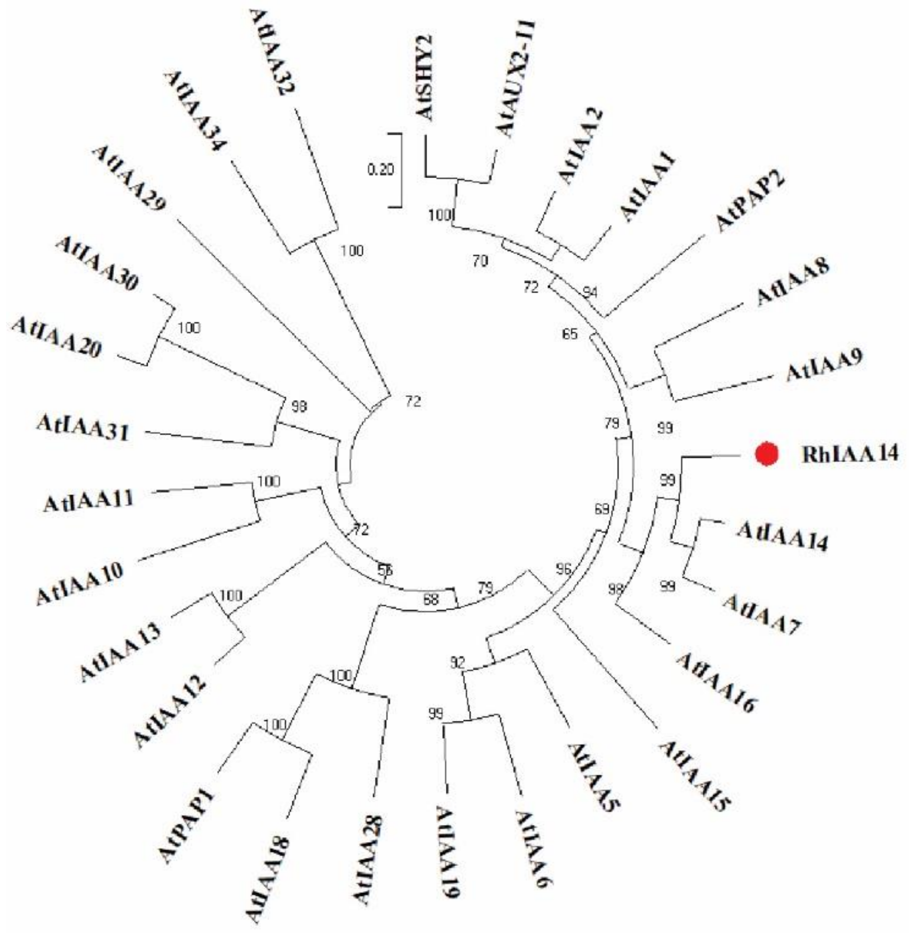

Figure S1. Phylogenetic analysis of RhIAA14 with Aux/IAA proteins from *Arabidopsis*. RhIAA14 was highlighted.

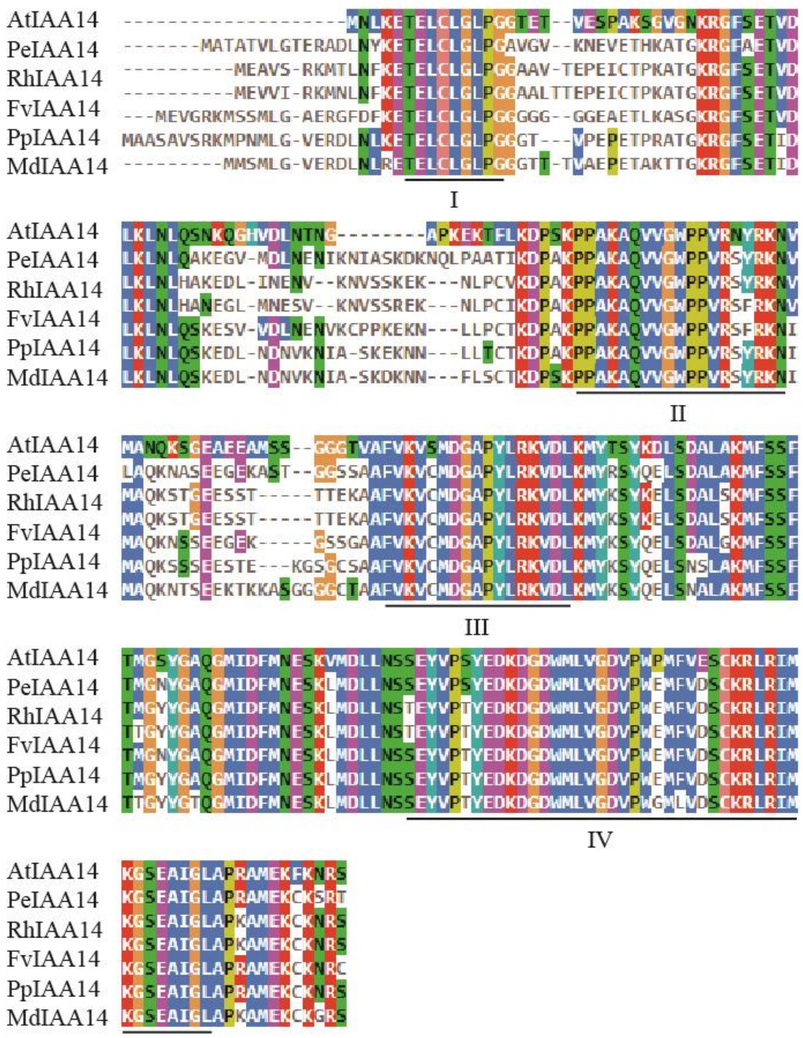

Figure S2. Multi-alignment of amino acid sequence of RhIAA14 with other plant IAA14 proteins. At, *Arabidopsis thaliana*; Pe, *Populus euphratica*; Rh, *Rosa hybrida*; Fv, *Fragaria vesca*; Pp, *Prunus persica*; Md, *Malus domestica*. Conserved amino acids were set to the same color. The underlines indicate the conserved domains (I–IV) of Aux/IAAs, respectively.

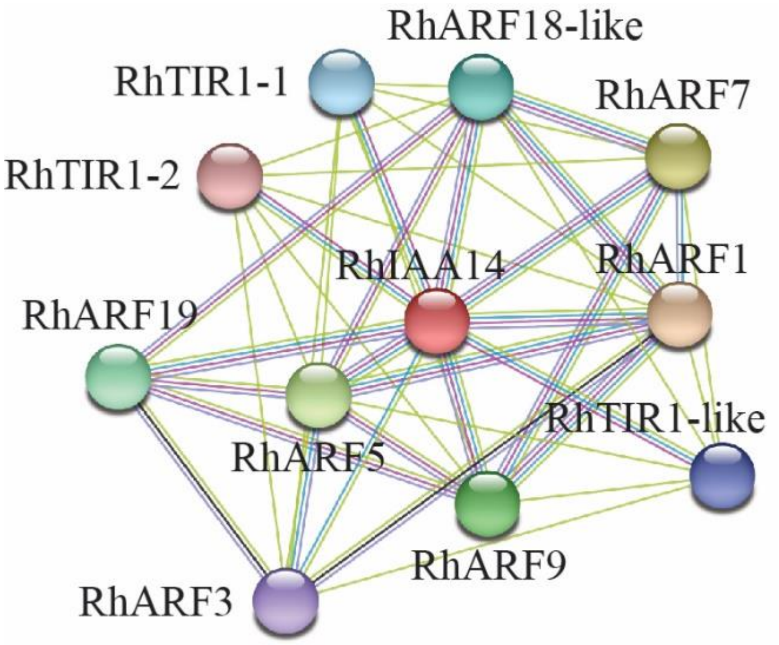

Figure S3. Predicted proteins interacting with RhIAA14.
